# Commentary: Can Inner Experience Be Apprehended in High Fidelity? Examining Brain Activation and Experience from Multiple Perspectives

**DOI:** 10.3389/fpsyg.2017.00360

**Published:** 2017-03-09

**Authors:** Henry D. Schlinger

**Affiliations:** Department of Psychology, California State University, Los AngelesLos Angeles, CA, USA

**Keywords:** pristine inner experience, introspection, consciousness, behavior, behavior analysis

Hurlburt et al. (2017) argue that they can potentially produce high-fidelity apprehensions of pristine inner experience that are radically non-subjective. In so doing, they claim that inner experience is an important topic and, contrary to almost unanimous scientific consensus, a method of introspection may be reliably used to directly apprehend it. In this and related articles (e.g., Hurlburt and Heavey, 2001; Heavy and Hurlburt, 2008), Hurlburt et al. reverse a general trend in psychology over the past 100 years toward studying behavior and away from studying subjective experience. In this commentary, I offer a logical and explicitly behavioristic (i.e., Skinnerian) critique of some of Hurlburt et al.'s points.

By “pristine experience,” Hurlburt et al. mean phenomena (e.g., thoughts, feelings, sensations, perceptions, etc.) that at a given moment appear directly “before the footlights of consciousness,” to use William James' words, which I take to mean when individuals become aware of the experience. Notwithstanding the problems inherent in defining the terms “thoughts,” “feelings,” “sensations,” and “perceptions,” what does it mean to become aware of something? There are likely many phenomena that occur when people speak of “awareness” or “consciousness.” For example, we speak of “consciousness” when organisms are awake, when they sense the environment, and when they talk about the environment and themselves (see Schlinger, 2008). The latter usage of “conscious” or “aware” seems most relevant in critiquing the concept of pristine experience. Likewise, what does it mean to say that someone “observes” or “apprehends” inner experience? The person is clearly not seeing or hearing the experience. We typically say that people “observe” inner experience when they talk (to themselves) about the experience. On this view, both observing and being aware of the experience are evidenced by the same behavior—talking about it. If by “observe” or “apprehend” we mean talking to oneself, then on the analogy of the observer effect in physics it may not be possible to separate the act of observing the inner experience from the actual experience. Finally, what do we mean by “inner experience”? The term obviously refers to something private. Hurlburt et al. offer no operational definitions of these terms and, without knowing exactly what they are referring to, we must be skeptical about their suggestion that we can ever apprehend inner experience or that the experience can be pristine.

By “radically non-subjective,” Hurlburt et al. mean that the inner experience is “not the result of opinion or impression but instead is directly apprehendable, as Skinner and the behaviorists required…” (p. 3). However, this statement misrepresents not only what behaviorists require, but what all scientists require: that phenomena be observable, at least in the initial, inductive stages of science. Although, reports of inner experience are objective, that is, they can be observed by others, the inner experience itself cannot be; it is, after all, “observed” by only one person in the sense that he or she labels or describes it either overtly or covertly. To further muddy the waters, descriptions of inner experience are metaphors because when we say that we covertly (or “innerly”) “see” or “hear,” we are not reacting to actual visual and auditory stimuli. In addition to the subjectivity of the reports, the problem is they are inherently unreliable (see Nisbett and Wilson, 1977; Johansson et al., 2006).

Hurlburt et al. suggest that one way to solve the problem of subjectivity and to demonstrate faithful apprehensions of pristine inner experience is to correlate brain activation, for example, by using fMRI, “with a variety of experiential perspectives” (p. 1). The perspective they describe is an introspective method they call Descriptive Experience Sampling (DES) in which individuals are instructed to describe their inner experience when they hear a randomly presented beep. In one example, Hurlburt et al. state that Susan “clearly innerly sees her boyfriend and his mother on a hillside next to the lake (much like she had actually seen them yesterday)” (p. 3). But that would be impossible because the boyfriend and mother were not actually there. Hurlburt et al. go on to say that although Susan's experience was private, whether “Susan was or was not innerly seeing her boyfriend and his mother is *not* a matter of subjective impression but of (Susan's radically non-subjective) direct apprehension” (p. 4). But Susan's experience *was* subjective. If by “apprehend” we mean perceive, and by “perceive” we mean to react to, then maybe Susan did react to some private experience. Because the experience was private, we can neither verify nor completely trust what she claimed to be reacting to.

Hurlburt et al. admit that “there is no well-developed scientific strategy to evaluate a claim about the fidelity of apprehensions/descriptions of private experience” (p. 5). At issue, then, is whether the DES attempts at fidelity are credible. And there is at least some reason to be skeptical of data from fMRI correlational studies (see Eklund et al., 2016). The critical questions are whether this elaborate and expensive attempt to tackle the perennial problem of conscious experience that has plagued psychologists (and philosophers) for centuries can achieve the results Hurlburt et al. believe it can, and whether it is worth the time and money.

Hurlburt et al. also claim that “first-person accounts are scientifically acceptable within science,” that “inner experience is indeed a defining aspect of the human condition, and psychological science must use first-person reports of inner experience…” (p. 2). To begin with, first-person accounts in the natural sciences are not scientifically acceptable. Those sciences are characterized by the discovery of universal laws of nature. Even though many psychologists view their discipline as a social science, I believe that psychologists should aspire to be natural scientists in seeking universal laws of human behavior. And first-person accounts are not going to get them there. Moreover, humans cannot be the only species with inner experience, although we are the only species that can talk about it (see Schlinger, 2009; Morin, 2012). Thus, talking about inner experience may be a defining aspect of humans, and that would be scientifically acceptable to study because talking is behavior that can be directly observed and measured.

A more parsimonious approach is to consider much of the inner experience of verbal humans as an epiphenomenon of human language. Thus, by studying human language scientifically—what people say and why they say it—by inference we might understand at least one type of inner experience—inner speech—just as natural scientists infer unobserved events only after studying observed events (Schlinger, 1998). Hurlburt et al. however, state that some individuals either rarely or never talk to themselves; this is their “pristine experience.” Just because some individuals report rarely talking to themselves does not mean that is so. A simpler explanation is that they are not aware of doing so. In other words, some people may never have learned to label their covert self-talk.

The alternative to studying inner experience is to study behavior and abandon, once and for all, psychologists' obsessive interest in mental events. Evolutionarily and psychologically, it is what an organism does, that is, how it behaves, that determines whether it will successfully navigate its environment and live long enough to pass on its genes. Because reports of inner experience will always be plagued by subjectivity and unreliability, a better use of time and money would be to study observed behavior as a subject matter. When Skinner (1977) wrote that “The appeal to cognitive states and processes is a diversion which could well be responsible for much of our failure to solve our problems” (p. 10), he meant that human problems are behavioral and that focusing on mental states is an obstacle to understanding and changing our behavior.

## Author contributions

The author confirms being the sole contributor of this work and approved it for publication.

### Conflict of interest statement

The author declares that the research was conducted in the absence of any commercial or financial relationships that could be construed as a potential conflict of interest.
